# Supplementary material for: Identification of a Novel Yersinia enterocolitica Strain from Bats in Association with a Bat Die-Off That Occurred in Georgia (Caucasus)
Source: Microorganisms. 2020 Jul 4;8(7):1000. doi: 10.3390/microorganisms8071000 (PMC7409352; doi:10.3390/microorganisms8071000)
Supplement: Supplementary file 1 [file microorganisms-08-01000-s001.pdf]

Identification of a Novel *Yersinia enterocolitica* Strain from Bats in Association with a Bat Die-off that Occurred in Georgia (Caucasus)

Tata Imnadze [1,2,†], Ioseb Natradze [3,†], Ekaterine Zhgenti [1], Lile Malania [1], Natalia Abazashvili [1], Ketevan Sidamonidze [1], Ekaterine Khmaladze [1], Mariam Zakalashvili [1], Paata Imnadze [1,2], Ryan J. Arner [4], Vladimir Motin [5] and Michael Kosoy [6,*]

supplemental

Supplement Table 1. Presence of the classical virulence-associated genes of *Yersinia enterocolitica* in the bat strains from Georgia.

Gene.	Function .	Presence..
ystB.	Heat-stable enterotoxin type B.	+..
invA .	Invasin .	+..
myfA.	Fibrillious adhesin .	+..
ymoA.	Modulator of expression for virulence functions .	+..
hreP.	In vivo-expressed protease .	+..
febBDGC/fes/fepA.	Enterochelin utilization cluster.	+..
ltxA .	Leukotoxin.	+..
rtxA.	RtxA-like putative leukotoxin .	+..
hemPR/hmuVSTU.	Direct heme uptake system.	+..
yax.	Cytotoxin YaxAB.	+..
ylpA.	Phospholipase.	+..
flg.	Flagella cluster I.	+..
pla.	Protease phospholipase A.	+..
arsCBR.	Arsenic cluster .	+..
yts1 .	Type 2 secretion.	+..
sctEJ/yscNU.	Chromosomal Type 3 secretion apparatus.	+..
virF.	Virulence regulon transcriptional activator .	+..
pYV plasmid.	Virulence plasmid encoding Type 3 secretion .	-..
ystA.	Heat-stable Enterotoxin type A.	-..
ail .	Adhesin .	-..
vapC.	Virulence-associated protein.	-..
vagC.	Virulence-associated protein.	-..
tccC.	Insecticidal toxin.	-..

The reported virulence factors are selected according to Batzilla et.al. [16].

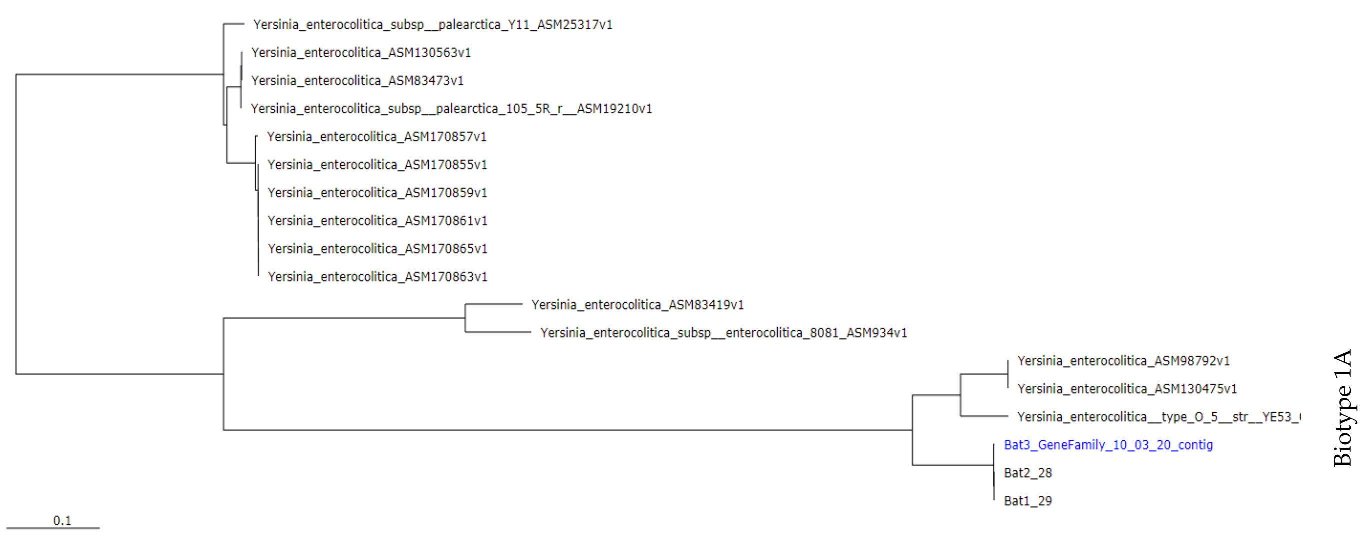

Supplement Figure S1. SNP based maximum likelihood phylogeny tree (RAxML) of three *Y. enterocolitica* bat isolates and *Y. enterocolitica* reference strains from a drop-down list of RefSeq complete genomes (EDGE).

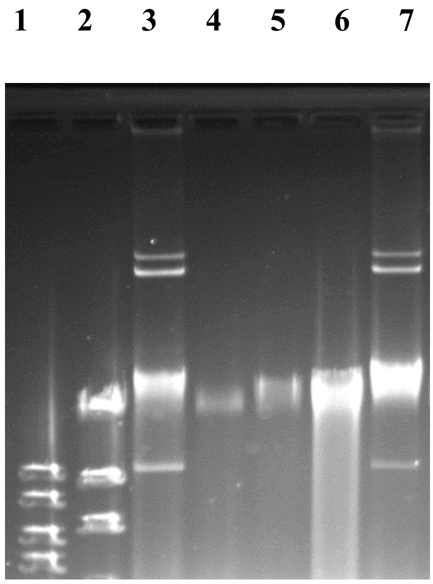

Supplement Figure S2. Plasmid profiles of the *Y. enterocolitica* strains obtained by Kado & Liu method. Lanes: 1. Molecular size standards (1 kb DNA Ladder, New England Biolabs); 2. Molecular size standard phage Lambda (Hind III); 3, 7. *Y. pestis* vaccine strain EV 76: 4, *Y. enterocolitica* BAT-1; 5, *Y. enterocolitica* BAT-2; 6. *Y. enterocolitica* BAT-3. *Y. pestis* vaccine strain EV76 contains plasmids with molecular weight 101, 70.5, and 9.5 kb.

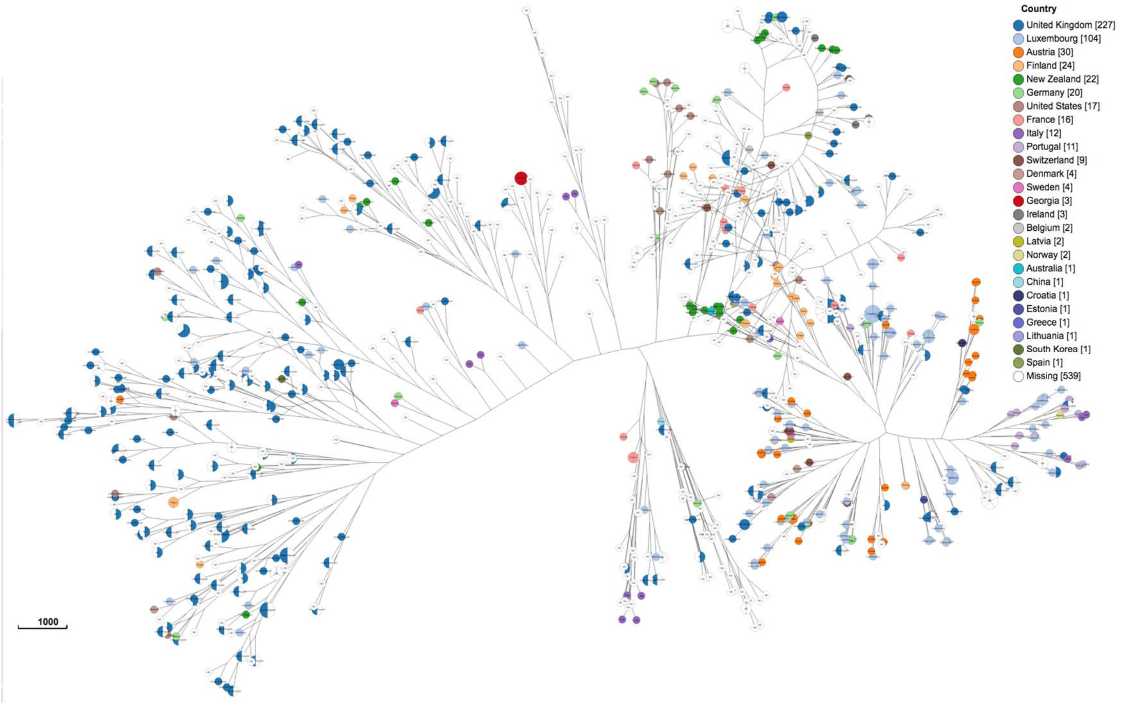

Supplement Figure S3. Results of core genome multi-locus sequence typing in Enterobase showing the position of *Y. enterocolitica* Bat1, 2, and 3 isolates in the context of worldwide *Y. enterocolitica* distribution. The Bat isolates are labelled “Georgia” and are in red color. ND- Not Determined, origin of isolate is missing in the database.
